# Antenatal Detection Rate of Foetal Growth Restriction in Primary Healthcare Centres: A Service Evaluation From Dammam, Saudi Arabia

**DOI:** 10.7759/cureus.105222

**Published:** 2026-03-14

**Authors:** Hind Al-Blowi, Abdallah D. AlKhathami, Neama Meriki, Thamer Metab Altherman, Fawziah G. Almalki, Areej M Alqahtani, Hanan Mahfouz Alghamdi, Alaa Al-Ajmi, Abrar Yousef Almarzooq

**Affiliations:** 1 Department of Ultrasound, Dammam Health Network, Dammam, SAU; 2 Department of Community Medicine, Dammam Health Network, Dammam, SAU; 3 Department of Obstetrics &amp; Gynecology, College of Medicine, King Saud University, Riyadh, SAU; 4 Department of Obstetrics &amp; Gynecology, Dammam Health Network, Dammam, SAU; 5 Department of Obstetrics &amp; Gynecology, Maternity and Children Hospital, Dammam, SAU; 6 Department of Family Medicine, Eastern Health Cluster, Dammam Health Network, Dammam, SAU; 7 Primary Health Care Centre, Dammam Health Network, Dammam, SAU

**Keywords:** antenatal screening, foetal growth restriction, perinatal outcomes, primary health care, ultrasonography (usg)

## Abstract

Introduction

We aimed to evaluate the antenatal detection of foetal growth restriction (FGR) in pregnant women receiving care at primary healthcare centres (PHCCs) in Dammam, Saudi Arabia.

Methods

This prospective cohort study was conducted as a service evaluation of routine antenatal care pathways in PHCCs in Dammam, Saudi Arabia. Pregnant women who underwent dating scans between January and May 2024 were enrolled and followed up until December 2024 to identify and detect FGR using standardised ultrasonography based on the Delphi consensus criteria.

Results

Among 210 pregnancies with complete birth outcomes, 19 neonates (9.0%; 95% confidence interval (CI): 5.9%-13.7%) were identified as having FGR. Of these, 12 (63.2%; 95% CI: 41.0%-80.9%) were detected antenatally at PHCCs using the Delphi consensus criteria, and seven (36.8%) were identified postnatally, corresponding to women who missed the scheduled third-trimester ultrasonography examination (growth scan) at 32-36 weeks. Most participants completed the follow-up (90.5%; 210/232; 95% CI: 86.1%-93.7%).

Conclusion

FGR was identified in 9% of pregnancies within the PHCCs. However, a considerable proportion of FGR cases remain undiagnosed antenatally, largely because of missed third-trimester growth scans, which underlie these undetected cases and underscore the need for improved adherence to scheduled ultrasonography. PHCCs can be the first line of surveillance for FGR when supported by structured systems and trained staff.

## Introduction

Foetal growth restriction (FGR) is a major challenge in obstetric care owing to its strong association with perinatal morbidity, mortality, and long-term neurodevelopmental and metabolic complications [1]. FGR affects approximately 10% of pregnancies [2] and is difficult to consistently detect, with global reports indicating suboptimal antenatal detection rates [3]. This condition arises from multifactorial aetiologies, including maternal, placental, and foetal factors as well as broader social determinants such as maternal age, socioeconomic status, and healthcare access [1]. Therefore, the accurate assessment of foetal growth is a fundamental objective of antenatal care and is critical for improving pregnancy outcomes [1].

Studies conducted in Saudi Arabia have primarily focused on hospital-based populations to investigate maternal risk factors and neonatal outcomes [4,5]. A recent study from Jazan, Saudi Arabia, reported a community-based FGR prevalence of 1.9% [6], although it did not focus on pregnancies managed at primary healthcare centres (PHCCs). Consequently, national-level evidence on the prevalence and detection of FGR in PHCC settings remains limited.

Health system reforms under Saudi Arabia’s Vision 2030 have led to the creation of regional health clusters aimed at enhancing the quality of care and reducing hospital burden [7]. Within this framework, the Eastern Health Cluster introduced a unique Safe Birth Pathway that redefined the responsibilities of PHCCs. In this model, intermediate-risk pregnancies are managed outside hospitals at PHCCs by board-certified obstetricians until 36 weeks of gestation, whereas low-risk pregnancies are managed by primary healthcare physicians in accordance with the national PHCC model [8]. This expanded role of the PHCC has been implemented in Eastern Province, where the Safe Birth Pathway was introduced under the regional health cluster framework. This model differs from that described by Munshi et al. [9] in which PHCCs were limited to managing low-risk pregnancies, with intermediate- and high-risk cases referred to hospitals.

The present study focused on FGR because it is a leading risk factor for stillbirth [10]. Although early detection does not eliminate all perinatal risks, it enables close monitoring and timely intervention, which are critical for improving outcomes [11]. However, missed diagnoses are common and often linked to limitations in routine antenatal screening, such as misinterpretation of estimated foetal weight (EFW) and inadequate use of symphysis-fundal height measurements [12].

The Safe Birth Pathway guides antenatal care delivery under Saudi Arabia’s national maternal care protocol, which includes risk stratification and referral of PHCCs to hospitals. Although it was designed to standardise care and reduce preventable adverse outcomes, its current structure does not require on-site ultrasound for PHCCs. Therefore, when FGR is suspected, patients must be referred to a hospital for further evaluation, which may lead to delays in diagnosis and timely interventions.

The Eastern Province has been at the forefront of healthcare reform with the establishment of a dedicated regional health cluster that expands antenatal services within PHCCs. As part of this expansion, a pilot ultrasound clinic was established in Dammam City to evaluate the readiness of PHCCs to detect FGR, as early detection is the cornerstone of follow-up, as emphasised by Atallah et al. [12].

This article was previously posted to Research Square (DOI: 10.21203/rs.3.rs-8271469/v1) on December 8, 2025.

## Materials and methods

Study design and setting

This prospective observational cohort study was conducted as a service evaluation at PHCCs in Dammam, Saudi Arabia. Participants were recruited between January and May 2024, and follow-up was extended through scheduled ultrasonographic assessments and postnatal outcome collection until December 2024.

Participants

Between January and May 2024, all pregnant women in their first trimester attending 22 PHCCs in Dammam were referred to a newly established central ultrasound clinic for routine gestational age assessment (dating scan) in accordance with a standardised protocol introduced as part of the Safe Birth Pathway. A total of 418 women who underwent routine first-trimester scans were included in the study. After receiving a detailed explanation of the study objectives and procedures, 232 participants provided written informed consent.

The inclusion criteria were as follows: (1) first-trimester pregnancy at the time of recruitment, (2) confirmed singleton pregnancy, and (3) normal second-trimester foetal anomaly scan. The exclusion criteria were known foetal congenital anomalies and multiple gestations.

Foetal anomaly scans were not conducted at the participating PHCCs but were instead performed at a designated maternity hospital between 18 and 22 weeks of gestation. The confirmation of a normal anomaly scan was based on hospital reports submitted by the participants during follow-up visits. Participants who did not have a documented anomaly scan report were initially retained in the study. However, if a major congenital anomaly was subsequently identified postnatally, the case was excluded from the analytical dataset in accordance with predefined exclusion criteria. This ensured that all included pregnancies were free of structural anomalies, either antenatally or postnatally, thereby maintaining consistency in the study sample.

As an accurate assessment of foetal growth requires reliable gestational dating, this study included only first-trimester pregnancies. This approach ensures a consistent baseline for evaluating foetal size and detecting growth restrictions. However, as recruitment was limited to women presenting early in pregnancy and attending routine dating scans, the findings may not be generalisable to all pregnant women receiving care at PHCCs, particularly those who present later in gestation or are not referred for early ultrasonography. This dating clinic is planned to remain operational beyond the study period, providing an ongoing system for accurate gestational age assignment across all PHCCs and enabling future quality improvements and research initiatives.

Sample size calculation

The required sample size was calculated using the following formula:

$n=\frac{Z^{2}\times p\left( 1-p \right)}{d^{2}}$

Assuming an expected FGR prevalence of 10% (P = 0.10), 95% confidence level (Z = 1.96), and 5% margin of error (d = 0.05), the minimum required sample size was 139 participants. The recruitment target was increased to account for potential losses to follow-up and incomplete data. Ultimately, 232 women were enrolled, which reduced the effective margin of error to approximately 3.86% and improved the precision of the prevalence estimates.

Calculations were performed using a standard statistical formula and verified using the OpenEpi online tool (www.openepi.com). OpenEpi is a publicly available, free online tool [13].

Ultrasonography assessment protocol

Each participant underwent two standardised ultrasonography examinations: the first between 24 and 31 weeks and the second between 32 and 36 weeks of gestation. At the time of the study, the participating PHCCs did not have on-site ultrasound clinics. A central ultrasound service was established as part of this project to provide dating scans as part of routine care, whereas growth scans were incorporated exclusively for research purposes to evaluate antenatal FGR detection. Upon completion of the study, only the dating scan service was retained as part of routine antenatal care, and growth scans were discontinued.

These assessments enable the identification of both early- and late-onset FGR according to the Delphi consensus classification [14]. The EFW was calculated using Hadlock’s three-parameter formula (head circumference, abdominal circumference, and femur length) [15]. FGR was diagnosed based on the Delphi consensus criteria, incorporating the following biometric measurements and Doppler indices: umbilical artery pulsatility index (PI > 95th percentile), middle cerebral artery PI (<5th percentile), and cerebroplacental ratio (<5th percentile).

All ultrasonography scans were performed by the principal investigator, a board-certified consultant obstetrician trained and certified in obstetric ultrasonography, using a Samsung Hera W9 instrument (Samsung Electronics Co., Ltd., Suwon, South Korea) to ensure consistency and reliability across cases. All antenatal FGR diagnoses were prospectively made by the principal investigator during scheduled assessments and documented in participants’ medical records.

Referral and follow-up

Participants with FGR were referred to a maternity hospital for further management. Neonatal outcomes, including birth weight and sex, were obtained from the medical records of the PHCCs and maternity hospitals. For participants who delivered outside the PHCC network, structured telephone interviews were conducted to obtain neonatal data. During these calls, participants were invited to share brief feedback on their antenatal care experience, providing additional qualitative insights into the barriers to and facilitators of follow-up.

Antenatal detection was defined as any case in which the participant was referred to a maternity hospital during pregnancy with a documented suspicion of FGR and the diagnosis was later confirmed after birth based on either birth weight below the third percentile or birth weight below the 10th percentile in combination with abnormal Doppler findings. Cases were classified as undetected antenatally if FGR was not identified during antenatal assessments, either because the participant did not return for follow-up growth scans or because the condition was not recognised at the time of scanning. In these cases, FGR was retrospectively diagnosed based on the postnatal data.

Data management and statistical analysis

This analysis focused on foetal outcomes, specifically the presence or absence of FGR. Maternal risk factors were excluded from the analysis.

The diagnosis of FGR was confirmed postnatally if the newborn’s birth weight was below the 10th percentile with abnormal Doppler findings documented during the antenatal growth scan or if the birth weight was below the third percentile, regardless of Doppler results. For participants who did not complete the follow-up, FGR was considered present if birth weight was below the third percentile. Birth weight data were obtained from hospital records or maternal reports of delivery cases outside the PHCC network.

All data were anonymised before entry, processed using Microsoft Excel (Microsoft Corporation, Redmond, WA, USA) for cleaning and validation, and imported into SPSS version 27 (IBM Corp., Armonk, NY, USA) for statistical analysis. Descriptive statistics (frequencies and percentages) were used to summarise the outcomes, including prevalence and antenatal FGR detection rates.

Prevalence estimates were calculated with 95% confidence intervals (CIs) using the exact binomial method. Participants with missing or unresolved outcome data were excluded from the final analysis. Sensitivity analyses were not performed because of the limited sample size and the observational design of the study.

Ethical considerations

Ethical approval was granted by the Institutional Review Board of the Dammam Medical Complex (approval number: IRB H05D107). All the procedures adhered to the ethical standards of the Declaration of Helsinki [16]. Participant confidentiality and anonymity were maintained using coded identifiers, and all participants retained the right to withdraw from the study at any stage with the assurance that their clinical care would not be affected.

## Results

Participant recruitment and follow-up

A total of 232 pregnant women participated in the study. Of these, 210 had complete birth outcome data and were included in the final analysis. The follow-up completion rate was 90.5% (210/232; 95% CI: 86.1%-93.7%). Twenty-two participants were excluded due to missing or unresolved birth outcome data. Among the excluded patients, four had documented congenital anomalies.

Maternal demographics

**Figure 1:**
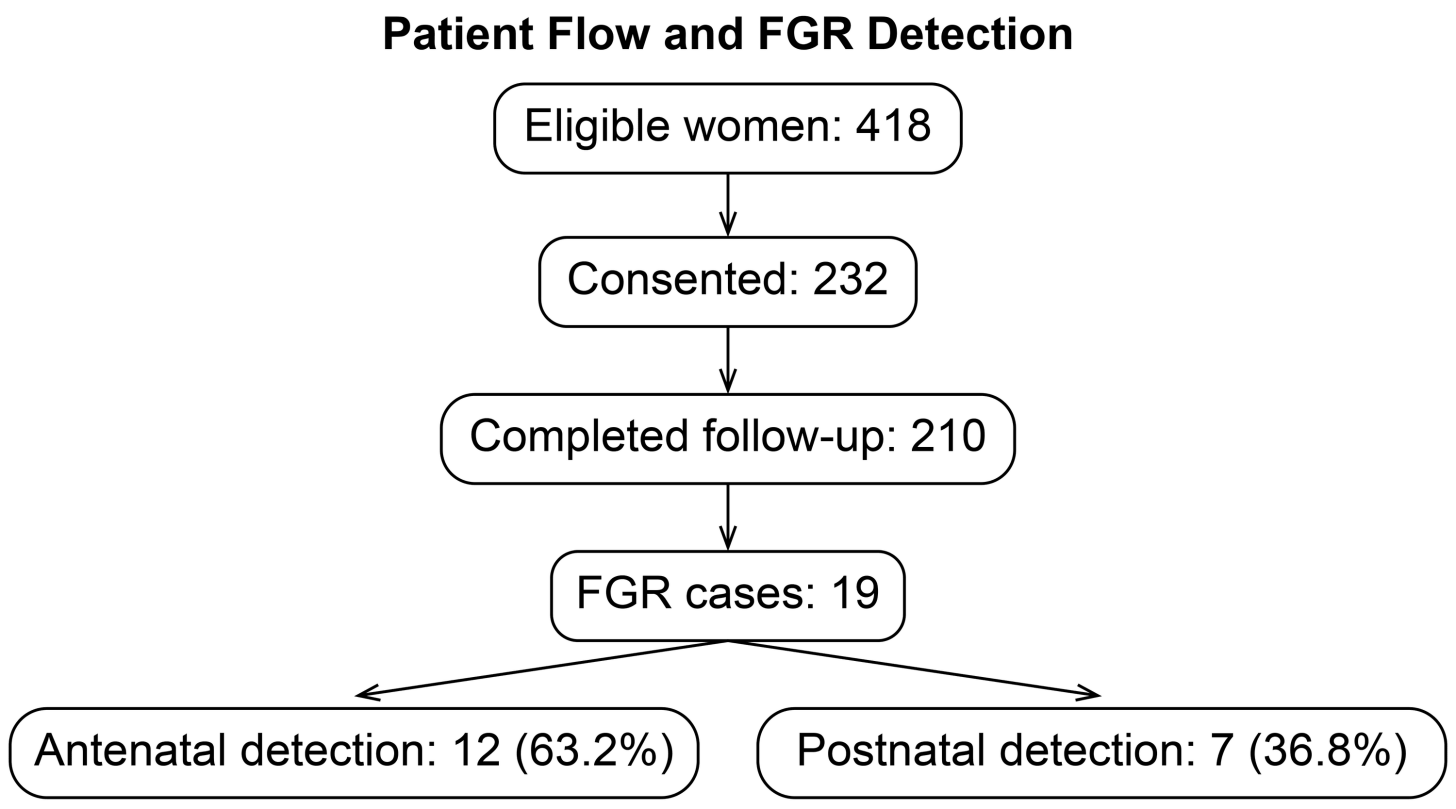
Study flowchart FGR: foetal growth restriction

Of the 232 pregnant women enrolled, 210 met the inclusion criteria and underwent ultrasonographic assessment (Figure 1). The mean maternal age was 28.7 years (standard deviation ± 5.2), with most participants (61.3%) aged between 25 and 34 years. Regarding parity, 105 women (45.7%) were nulliparous and 125 (54.3%) were multiparous. Key demographic and clinical characteristics are presented in Table 1.

**Table 1:** Demographic and clinical characteristics of the study participants (n = 210)

Characteristic	n (%)
Maternal age (years)	
<25	47 (22.4)
25-34	129 (61.3)
≥35	34 (16.3)
Parity	
Nulliparous	96 (45.7)
Multiparous	114 (54.3)

No statistically significant differences in age or parity were observed between participants with complete and those with missing outcome data (data not shown).

Prevalence and antenatal detection of FGR

Among the 210 pregnancies analysed, 19 (9.0%; 95% CI: 5.9%-13.7%) were identified as having FGR. Of these, 12 cases (63.2%; 95% CI: 41.0%-80.9%) were detected antenatally during scheduled ultrasonography at the PHCCs based on the Delphi consensus classification criteria [14]. The remaining seven cases (36.8%) were identified postnatally and classified as missed antenatal diagnoses.

All undetected cases occurred because participants missed their scheduled third-trimester growth scans. None of the participants who underwent the scan was incorrectly classified as having FGR.

Summary of birth outcome completeness and FGR detection

Tables 2, 3 summarise the completeness of birth outcome data and antenatal detection outcomes among pregnancies complicated by FGR, distinguishing between cases detected antenatally at PHCCs and those identified postnatally due to missed third-trimester ultrasonography.

**Table 2:** Completeness of birth outcome data Data source: study dataset

Outcome status	n	%
Complete birth outcome data	210	90.5
Missing outcome information (excluding anomalies)	18	7.8
Major congenital anomalies	4	1.7
Total	232	100.0

**Table 3:** Antenatal detection of foetal growth restriction (FGR) Data source: study dataset PHCCs: primary healthcare centres

Condition status	n	%
Diagnosed antenatally at PHCCs based on Delphi criteria [14]	12	63.2
Missed scheduled third-trimester ultrasonography	7	36.8
Total	19	100.0

Among the 19 patients with FGR, 12 were in the low-risk group and seven from the intermediate-risk group. Missed third-trimester scans occurred more frequently in the low-risk group (6/12, 50%) than in the intermediate-risk group (1/7, 14.3%). These findings underscore the importance of follow-up ultrasonographic appointments in improving the detection rate of FGR at the primary healthcare level, particularly in low-risk pregnancies.

## Discussion

The prevalence of FGR among women receiving care at PHCCs in Dammam, Saudi Arabia, was 9%, which is comparable to the global prevalence estimate of approximately 10% [2]. The antenatal detection rate was 63.2%. However, 36.8% of cases were diagnosed postnatally, primarily because of missed third-trimester growth scans.

Although the prevalence rate determined in our study is consistent with those of other international reports [2], a study from Jazan, Saudi Arabia, reported a significantly lower prevalence of 1.9% [6]. This discrepancy may be explained by the differences in the study population, methodology, or diagnostic criteria. Other studies conducted in Riyadh and Jeddah have been largely hospital-based, focusing primarily on maternal risk factors and neonatal outcomes rather than on population-level prevalence [4,5]. Unlike these hospital-centred studies, our study evaluated FGR detection within PHCCs, highlighting the system-level challenges specific to primary care settings.

Evidence on antenatal FGR detection rates within PHCC settings remains limited. In our study, the antenatal detection rate was 63.2%, which compares favourably with both hospital-based data and dedicated cohort studies. For instance, in the French REPERE cohort [3], antenatal detection rates for small-for-gestational-age foetuses remained below 50%, underscoring diagnostic challenges, even in high-resource settings. Similarly, a recent study in South Africa demonstrated the feasibility of implementing Doppler-based screening protocols for PHCCs between 28 and 34 weeks of gestation to identify at-risk foetuses [17]. These findings support the view that, with appropriate protocolisation and capacity building, PHCC-based FGR detection, as demonstrated in our model, could improve the performance of hospital-based systems.

The relatively high proportion of missed FGR cases in this study could be primarily attributed to the absence of routine third-trimester growth scans within PHCCs. These challenges are particularly pronounced in PHCCs that lack ultrasound capability. In Dammam, a major city within the Eastern Health Cluster, none of the 22 PHCCs currently operates on-site ultrasound clinics staffed with qualified personnel trained in foetal growth assessment. Such clinics require both functional ultrasound equipment and a specialist trained in obstetric ultrasonography to ensure accurate detection of FGR. The absence of these services significantly limits access to structured foetal growth evaluations, particularly for low-risk pregnant women managed entirely within PHCC settings.

Informal feedback from participants after delivery suggests divergent patterns in care-seeking behaviours between the risk groups. Intermediate-risk women managed by obstetricians generally benefit from structured appointments. In contrast, some low-risk women managed by primary care physicians reported challenges, such as self-booking requirements, inconsistent provider availability, and limited appointment slots. Although not systematically collected or quantified, these anecdotal insights highlight potential system-level barriers to patient engagement and continuity of care, which are recognised as key determinants of antenatal care adherence [18,19].

Thus, despite Vision 2030-driven expansion of PHCC responsibilities and the Safe Birth Pathway’s emphasis on risk stratification and early detection, gaps remain in service delivery. Specifically, the absence of routine third-trimester ultrasound limits timely FGR diagnosis and undermines the effectiveness of these reforms. This indicates the need to reevaluate structural policies and resource allocation within the PHCC system to align actual service delivery with national policy goals.

A major strength of this study was its prospective cohort design, which allowed for a systematic assessment of FGR in real-world PHCC settings [20]. This study also achieved a high follow-up completion rate of 90.5%, which enhanced the reliability of prevalence and detection estimates. Finally, the implementation of standardised ultrasonography using the Delphi consensus criteria [14], performed by a certified consultant obstetrician, ensured diagnostic accuracy and minimised variability.

In addition, this study investigated FGR identification specifically at the PHCC level rather than in hospital-based settings/populations. By focusing on system-level performance rather than individual maternal risk factors, this study provides unique insights into the feasibility and challenges of implementing structured foetal growth surveillance in PHCCs.

This study has some limitations. First, the relatively small number of confirmed FGR cases may limit the generalisability of our results to a broader population [21]. Second, although maternal and foetal risk factors were included in the questionnaire, they were not analysed because the primary aim was to assess system-level detection performance rather than individual predictors. Third, qualitative observations of care-seeking behaviour were derived from informal, unstructured postnatal conversations rather than systematic data collection and should therefore be interpreted as exploratory insights only. Additionally, the sample was derived from women referred for dating ultrasonography rather than from all PHCC attendees, potentially introducing a selection bias [22]. Recruitment was non-random and limited to a defined period and a single city, which may limit generalisability to other PHCCs or regions [23]. Finally, all ultrasound assessments were performed by a single investigator. Although this ensured standardisation, it may have introduced observer bias despite internal consistency.

Our findings highlight the need to strengthen antenatal care pathways in PHCCs. First, the research findings present a strong case for the integration of routine third-trimester growth scans into national antenatal care protocols, as evidence shows that universal third-trimester ultrasound substantially improves FGR [24]. Second, a more efficient healthcare model could establish a dedicated ultrasound clinic within each PHCC network (e.g., one clinic serving a single-city network). All pregnant women, regardless of risk classification (low or intermediate risk), were referred to these clinics for dating and routine third-trimester growth scans between 32 and 36 weeks of gestation (anomaly scans should be performed in hospital-based settings). Network-based clinics should be staffed with trained sonographers competent in growth and Doppler assessments and authorised to directly refer patients to hospitals in cases of abnormal results, thereby minimising delays in identifying FGR. In suspected FGR cases with normal Doppler findings, the affected women were observed and evaluated on a designated day by a visiting obstetrician, ensuring timely evaluation without unnecessary hospital referrals. Third, establishing dedicated antenatal clinics led by certified family physicians within PHCCs would ensure that antenatal care is delivered in a setting specifically designed for pregnancy follow-up, rather than in general outpatient clinics. Such dedicated clinics could enhance continuity of care and improve the overall patient experience. Finally, patient engagement strategies such as reminder systems, flexible appointment scheduling, and clear care pathways should be implemented to reduce barriers to follow-up. These recommendations align with the World Health Organisation guidelines, which emphasise flexible scheduling, reminders, and continuity of care as key strategies to improve antenatal attendance and promote a positive pregnancy experience [25]. The proposed system-level interventions are consistent with the National Health Transformation Goals and can significantly improve maternal and neonatal outcomes in Saudi Arabia.

Future studies should explore the scalability and cost-effectiveness of introducing routine third-trimester growth scans at the PHCC level, particularly in network-based ultrasound clinics. Multicentre studies across different regions of Saudi Arabia are needed to assess whether this model can be adapted nationwide and to evaluate its impact on maternal and neonatal outcomes. Further research is needed to investigate patient engagement strategies, such as digital reminders and streamlined appointment systems, to determine their effectiveness in improving follow-up adherence and detecting FGR [25]. Finally, qualitative studies focusing on the antenatal care experiences of pregnant women followed up in PHCCs may elucidate barriers and facilitators, informing the design of patient-centred interventions.

## Conclusions

Evidence on antenatal detection of FGR within PHCC settings in Saudi Arabia remains limited, despite ongoing national efforts to expand maternal services at the primary care level. This study addressed this gap by evaluating the antenatal detection rate and observed prevalence of FGR among pregnant women receiving care at PHCCs in Dammam, Saudi Arabia.

The prevalence of FGR among pregnant women receiving care at PHCCs was approximately 9%. Importantly, nearly two-thirds of the FGR cases were identified antenatally, including cases from both the low- and intermediate-risk groups, indicating that PHCCs play a meaningful role in identifying affected pregnancies. PHCCs can serve as the first line of detection and follow-up for FGR, underscoring the importance of investing in their capacity. Strengthening PHCCs with adequate equipment, trained personnel, and structured care pathways could enhance the early detection of FGR and establish these centres as active partners to alleviate the burden on hospital services and improve maternal and neonatal outcomes. These improvements are consistent with the objectives of Vision 2030 in Saudi Arabia, which emphasises strengthening primary healthcare services, prioritising preventive care, and enhancing maternal and neonatal health outcomes nationwide.
